# 1-(10-Bromo­anthracen-9-yl)-1*H*-imidazole

**DOI:** 10.1107/S160053681300891X

**Published:** 2013-04-10

**Authors:** Hon Man Lee, Hong-Jyun Lee

**Affiliations:** a National Changhua University of Education, Department of Chemistry, Changhua, 50058, Taiwan

## Abstract

In the title mol­ecule, C_17_H_11_BrN_2_, the planes of the anthracene ring system [maximum deviation from the mean plane = 0.036 (3) Å] and the imidazole ring form a dihedral angle of 85.14 (14)°. In the crystal, weak C—H⋯N and C—H⋯Br hydrogen bonds link the mol­ecules into double chains propagating along [01-1]. In addition, π–π stacking inter­actions between pairs of benzene rings are observed, with centroid–centroid distances of 3.7968 (17) and 3.8496 (16) Å.

## Related literature

For the preparation of the title compound, see: Lee *et al.* (2011 ▶). For the structure of a related compound, see: Boyer *et al.* (1993 ▶).
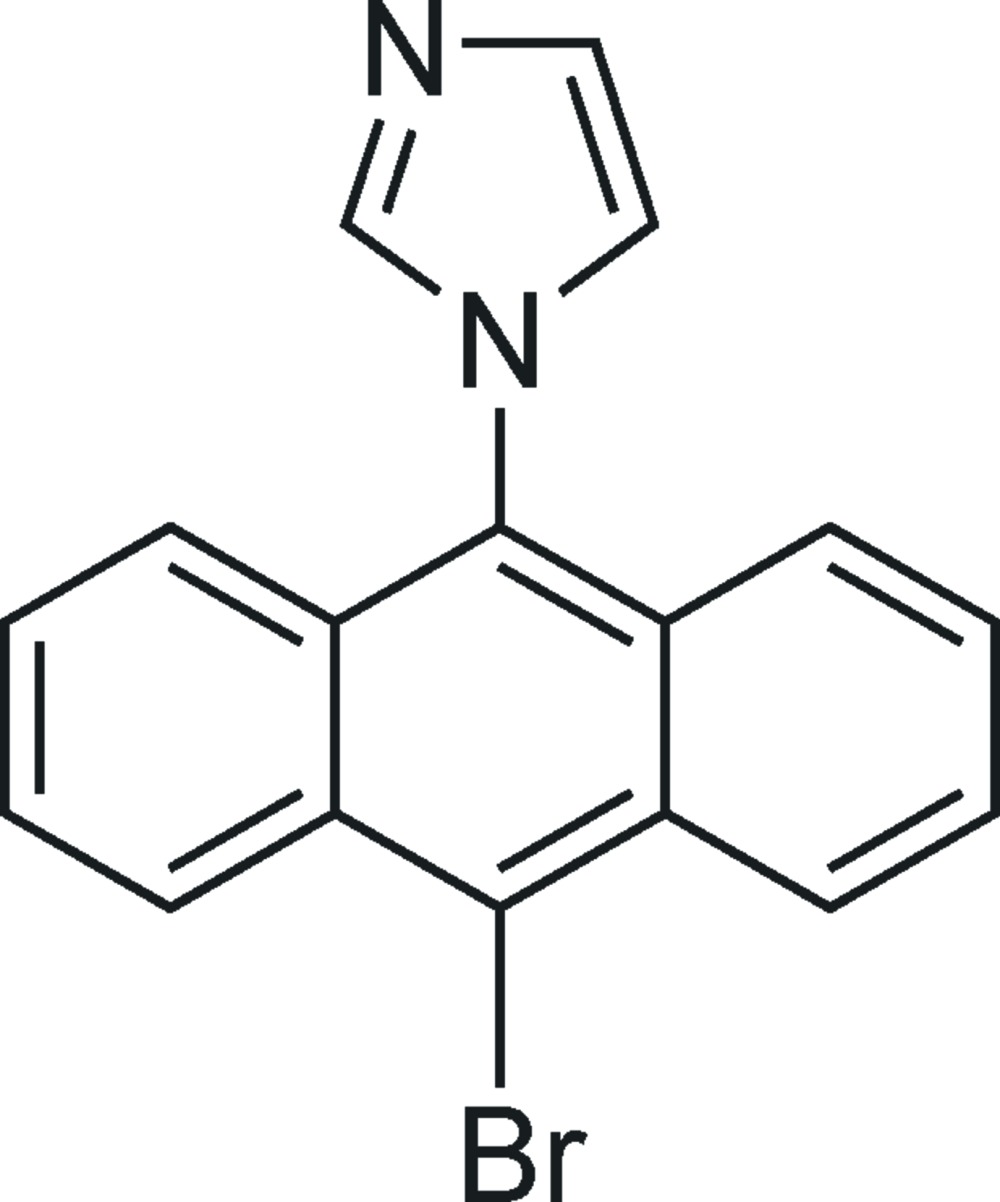


## Experimental

### 

#### Crystal data

C_17_H_11_BrN_2_
*M* *_r_* = 323.19
Triclinic, 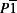

*a* = 8.1832 (2) Å
*b* = 8.8473 (2) Å
*c* = 9.5814 (2) Å
α = 75.355 (1)°
β = 81.624 (1)°
γ = 77.134 (1)°
*V* = 651.36 (3) Å^3^
*Z* = 2
Mo *K*α radiation
μ = 3.15 mm^−1^
*T* = 150 K
0.35 × 0.26 × 0.07 mm

#### Data collection

Bruker SMART APEXII diffractometer
Absorption correction: multi-scan (*SADABS*; Sheldrick, 2003 ▶) *T*_min_ = 0.406, *T*_max_ = 0.822
6236 measured reflections
2846 independent reflections
2562 reflections with *I* > 2σ
*R*_int_ = 0.017

#### Refinement

*R*[*F* ^2^ > 2σ(*F* ^2^)] = 0.032
*wR*(*F* ^2^) = 0.100
*S* = 1.11
2846 reflections
185 parameters
H atoms treated by a mixture of independent and constrained refinement
Δρ_max_ = 0.54 e Å^−3^
Δρ_min_ = −0.28 e Å^−3^

### 

Data collection: *APEX2* (Bruker, 2007 ▶); cell refinement: *SAINT* (Bruker, 2007 ▶); data reduction: *SAINT*; program(s) used to solve structure: *SHELXTL* (Sheldrick, 2008 ▶); program(s) used to refine structure: *SHELXTL*; molecular graphics: *DIAMOND* (Brandenburg, 2006 ▶); software used to prepare material for publication: *SHELXTL*.

## Supplementary Material

Crystal structure: contains datablock(s) I, global. DOI: 10.1107/S160053681300891X/lh5602sup1.cif

Structure factors: contains datablock(s) I. DOI: 10.1107/S160053681300891X/lh5602Isup2.hkl

Supplementary material file. DOI: 10.1107/S160053681300891X/lh5602Isup3.cml

Additional supplementary materials:  crystallographic information; 3D view; checkCIF report

## Figures and Tables

**Table 1:** Hydrogen-bond geometry (Å, °)

*D*—H⋯*A*	*D*—H	H⋯*A*	*D*⋯*A*	*D*—H⋯*A*
C3—H3⋯Br1^i^	0.95	2.86	3.687 (3)	147
C3—H3⋯N2^ii^	0.95	2.58	3.393 (4)	144

Symmetry codes: (i) 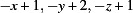; (ii) 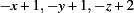.

**Fig. 1.**
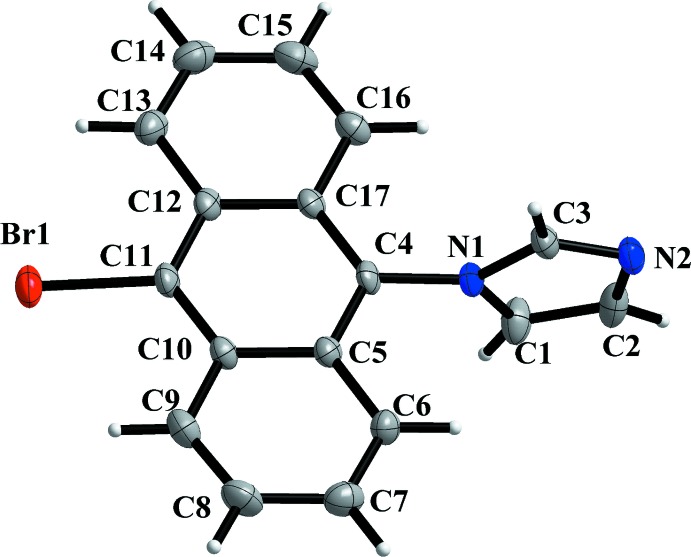
The molecular structure of the title compound, showing 50% probability displacement ellipsoids for the non-hydrogen atoms. The H atoms are dipicted by circles of an arbitrary radius.

**Fig. 2.**
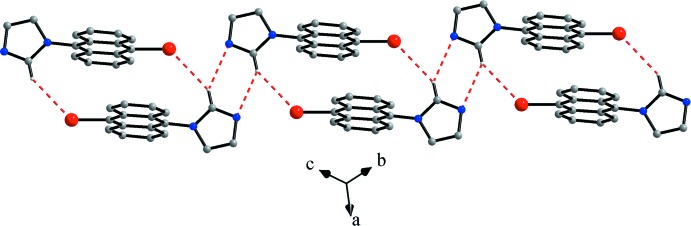
A view of a double chain displaying the hydrogen bonds as dashed lines. The viewing direction is along [1,1,1].

## supplementary crystallographic information

### Comment

The title compound is a side-product in the preparation of 9,10-di(1*H*-imidazol-1-yl)anthracene. The latter compound is a ditopic bis(imidazole) ligand which can form intriguing coordination polymers with transition metal ions (Lee *et al.*, *et al.* 2011). The title compound however, features only one imidazole moiety. 9-(10'-Bromo-9'-anthryl)carbazole is a structure in the literature which is related to the title compound (Boyer *et al.* 1993).

The molecular structure of the title compound is shown in Fig. 1. The anthracene ring system [maximum deviation from the mean plane = 0.036 (3)Å for C11] and imidazole ring form a dihedral angle of 85.14 (14) Å. In the crystal, weak hydrogen bonds of the type C—H···N and C—H···Br link the molecules into one-dimensional double chains propagating along [0, 1, -1] (Fig. 2). Double chains are further stablized by π–π stacking interaction between pairs of anthracene rings. The contact distances are 3.7968 (17) [*Cg*1···*Cg*1(-*x*, 2 - *y*, 1 - *z*)] and 3.8496 (16) Å [*Cg*1···*Cg*1(-*x*, 2 - *y*, 1 - *z*)]. *Cg*1 and *Cg*2 are the centroids of C12—C17 and C4/C5/C10—C12/C17, respectively.

### Experimental

The compound was isolated as a side-product in the preparation of 9,10-di(1*H*-imidazol-1-yl)anthracene (dia) (Lee *et al.*, 2011). Suitable crystals were obtained by slow evaporation of a methanol solution of the compound at room temperature.

### Refinement

The hydrogen atom upon C2 was located in a difference Fourier map and freely refined. All the other hydrogen atoms were positioned geometrically and refined as riding atoms, with C—H = 0.95 Å with *U*_iso_(H) = 1.2*U*_eq_(C).

### Crystal data

**Table:**

C_17_H_11_BrN_2_	*Z* = 2
*M**_r_* = 323.19	*F*(000) = 324
Triclinic, *P*1	*D*_x_ = 1.648 Mg m^−^^3^
Hall symbol: -P 1	Mo *K*α radiation, λ = 0.71073 Å
*a* = 8.1832 (2) Å	Cell parameters from 2906 reflections
*b* = 8.8473 (2) Å	θ = 2.4–27.9°
*c* = 9.5814 (2) Å	µ = 3.15 mm^−^^1^
α = 75.355 (1)°	*T* = 150 K
β = 81.624 (1)°	Block, colorless
γ = 77.134 (1)°	0.35 × 0.26 × 0.07 mm
*V* = 651.36 (3) Å^3^	

### Data collection

**Table:**

Bruker SMART APEXII diffractometer	2846 independent reflections
Radiation source: fine-focus sealed tube	2562 reflections with *I* > 2σ
Graphite monochromator	*R*_int_ = 0.017
ω scans	θ_max_ = 27.0°, θ_min_ = 2.2°
Absorption correction: multi-scan (*SADABS*; Sheldrick, 2003)	*h* = −10→10
*T*_min_ = 0.406, *T*_max_ = 0.822	*k* = −6→11
6236 measured reflections	*l* = −12→12

### Refinement

**Table:**

Refinement on *F*^2^	Primary atom site location: structure-invariant direct methods
Least-squares matrix: full	Secondary atom site location: difference Fourier map
*R*[*F*^2^ > 2σ(*F*^2^)] = 0.032	Hydrogen site location: inferred from neighbouring sites
*wR*(*F*^2^) = 0.100	H atoms treated by a mixture of independent and constrained refinement
*S* = 1.11	*w* = 1/[σ^2^(*F*_o_^2^) + (0.0608*P*)^2^ + 0.4219*P*] where *P* = (*F*_o_^2^ + 2*F*_c_^2^)/3
2846 reflections	(Δ/σ)_max_ < 0.001
185 parameters	Δρ_max_ = 0.54 e Å^−^^3^
0 restraints	Δρ_min_ = −0.28 e Å^−^^3^

### Special details

**Table:**

Geometry. All e.s.d.'s (except the e.s.d. in the dihedral angle between two l.s. planes) are estimated using the full covariance matrix. The cell e.s.d.'s are taken into account individually in the estimation of e.s.d.'s in distances, angles and torsion angles; correlations between e.s.d.'s in cell parameters are only used when they are defined by crystal symmetry. An approximate (isotropic) treatment of cell e.s.d.'s is used for estimating e.s.d.'s involving l.s. planes.
Refinement. Refinement of *F*^2^ against ALL reflections. The weighted *R*-factor *wR* and goodness of fit *S* are based on *F*^2^, conventional *R*-factors *R* are based on *F*, with *F* set to zero for negative *F*^2^. The threshold expression of *F*^2^ > σ(*F*^2^) is used only for calculating *R*-factors(gt) *etc*. and is not relevant to the choice of reflections for refinement. *R*-factors based on *F*^2^ are statistically about twice as large as those based on *F*, and *R*- factors based on ALL data will be even larger.

### Fractional atomic coordinates and isotropic or equivalent isotropic displacement parameters (Å^2^)

**Table:**

	*x*	*y*	*z*	*U*_iso_*/*U*_eq_	
Br1	0.29223 (4)	1.29770 (3)	0.32328 (3)	0.02958 (12)	
C1	0.0666 (4)	0.7593 (4)	0.9486 (3)	0.0298 (6)	
H1	−0.0397	0.8259	0.9291	0.036*	
C2	0.0985 (4)	0.6312 (4)	1.0596 (3)	0.0294 (6)	
C3	0.3352 (4)	0.6527 (3)	0.9386 (3)	0.0221 (5)	
H3	0.4520	0.6345	0.9072	0.027*	
C4	0.2461 (3)	0.8922 (3)	0.7399 (3)	0.0185 (5)	
C5	0.2927 (3)	1.0313 (3)	0.7525 (3)	0.0182 (5)	
C6	0.3187 (3)	1.0569 (3)	0.8880 (3)	0.0230 (5)	
H6	0.3047	0.9771	0.9739	0.028*	
C7	0.3633 (4)	1.1936 (4)	0.8968 (3)	0.0288 (6)	
H7	0.3781	1.2091	0.9883	0.035*	
C8	0.3875 (4)	1.3121 (3)	0.7700 (4)	0.0308 (6)	
H8	0.4218	1.4055	0.7767	0.037*	
C9	0.3622 (4)	1.2942 (3)	0.6381 (3)	0.0266 (6)	
H9	0.3776	1.3761	0.5543	0.032*	
C10	0.3129 (3)	1.1539 (3)	0.6238 (3)	0.0192 (5)	
C11	0.2836 (3)	1.1299 (3)	0.4912 (3)	0.0207 (5)	
C12	0.2413 (3)	0.9892 (3)	0.4780 (3)	0.0194 (5)	
C13	0.2162 (4)	0.9609 (3)	0.3427 (3)	0.0269 (6)	
H13	0.2307	1.0392	0.2559	0.032*	
C14	0.1720 (4)	0.8240 (4)	0.3366 (3)	0.0323 (7)	
H14	0.1561	0.8076	0.2457	0.039*	
C15	0.1494 (4)	0.7059 (3)	0.4631 (4)	0.0313 (7)	
H15	0.1166	0.6115	0.4571	0.038*	
C16	0.1741 (3)	0.7257 (3)	0.5940 (3)	0.0241 (6)	
H16	0.1598	0.6443	0.6784	0.029*	
C17	0.2214 (3)	0.8676 (3)	0.6065 (3)	0.0188 (5)	
H2	0.028 (4)	0.592 (4)	1.127 (4)	0.028 (9)*	
N1	0.2190 (3)	0.7738 (3)	0.8691 (2)	0.0197 (4)	
N2	0.2670 (3)	0.5647 (3)	1.0537 (3)	0.0255 (5)	

### Atomic displacement parameters (Å^2^)

**Table:**

	*U*^11^	*U*^22^	*U*^33^	*U*^12^	*U*^13^	*U*^23^
Br1	0.03324 (19)	0.02543 (17)	0.02217 (17)	−0.00272 (12)	−0.00137 (11)	0.00551 (11)
C1	0.0215 (14)	0.0357 (16)	0.0255 (15)	−0.0028 (12)	−0.0044 (11)	0.0041 (12)
C2	0.0327 (16)	0.0333 (15)	0.0203 (14)	−0.0138 (13)	−0.0029 (12)	0.0037 (12)
C3	0.0235 (13)	0.0176 (12)	0.0214 (13)	0.0005 (10)	−0.0061 (10)	0.0005 (10)
C4	0.0173 (12)	0.0156 (11)	0.0184 (12)	0.0014 (9)	−0.0049 (10)	0.0016 (10)
C5	0.0164 (12)	0.0174 (12)	0.0184 (12)	0.0019 (9)	−0.0045 (9)	−0.0024 (10)
C6	0.0243 (13)	0.0233 (13)	0.0193 (13)	−0.0003 (10)	−0.0058 (10)	−0.0025 (10)
C7	0.0279 (15)	0.0314 (15)	0.0287 (15)	0.0010 (12)	−0.0091 (12)	−0.0123 (12)
C8	0.0332 (16)	0.0218 (13)	0.0402 (17)	−0.0043 (11)	−0.0097 (13)	−0.0098 (12)
C9	0.0271 (14)	0.0175 (12)	0.0323 (15)	−0.0020 (11)	−0.0061 (12)	−0.0006 (11)
C10	0.0181 (12)	0.0155 (11)	0.0212 (13)	0.0003 (9)	−0.0052 (10)	−0.0003 (10)
C11	0.0208 (13)	0.0172 (12)	0.0179 (12)	0.0023 (10)	−0.0032 (10)	0.0026 (10)
C12	0.0170 (12)	0.0190 (12)	0.0179 (12)	0.0038 (9)	−0.0046 (9)	−0.0014 (10)
C13	0.0279 (14)	0.0283 (14)	0.0204 (13)	0.0046 (11)	−0.0063 (11)	−0.0043 (11)
C14	0.0329 (16)	0.0378 (16)	0.0277 (15)	0.0034 (13)	−0.0093 (12)	−0.0154 (13)
C15	0.0310 (16)	0.0240 (14)	0.0417 (18)	0.0012 (12)	−0.0110 (13)	−0.0141 (13)
C16	0.0225 (13)	0.0191 (12)	0.0296 (15)	−0.0009 (10)	−0.0059 (11)	−0.0046 (11)
C17	0.0175 (12)	0.0156 (11)	0.0204 (13)	0.0022 (9)	−0.0050 (10)	−0.0019 (10)
N1	0.0218 (11)	0.0175 (10)	0.0170 (10)	−0.0018 (8)	−0.0044 (9)	0.0012 (8)
N2	0.0345 (13)	0.0192 (11)	0.0208 (11)	−0.0053 (9)	−0.0076 (10)	0.0017 (9)

### Geometric parameters (Å, º)

**Table:**

Br1—C11	1.898 (3)	C7—H7	0.9500
C1—C2	1.350 (4)	C8—C9	1.362 (4)
C1—N1	1.377 (4)	C8—H8	0.9500
C1—H1	0.9500	C9—C10	1.431 (4)
C2—N2	1.372 (4)	C9—H9	0.9500
C2—H2	0.86 (4)	C10—C11	1.401 (4)
C3—N2	1.309 (4)	C11—C12	1.403 (4)
C3—N1	1.366 (3)	C12—C17	1.432 (4)
C3—H3	0.9500	C12—C13	1.432 (4)
C4—C17	1.400 (4)	C13—C14	1.356 (4)
C4—C5	1.404 (4)	C13—H13	0.9500
C4—N1	1.431 (3)	C14—C15	1.405 (5)
C5—C6	1.426 (4)	C14—H14	0.9500
C5—C10	1.438 (4)	C15—C16	1.358 (4)
C6—C7	1.364 (4)	C15—H15	0.9500
C6—H6	0.9500	C16—C17	1.430 (4)
C7—C8	1.411 (5)	C16—H16	0.9500
			
C2—C1—N1	106.2 (3)	C11—C10—C9	123.4 (2)
C2—C1—H1	126.9	C5—C10—C9	118.1 (2)
N1—C1—H1	126.9	C10—C11—C12	122.8 (2)
C1—C2—N2	110.7 (3)	C10—C11—Br1	118.82 (19)
C1—C2—H2	128 (2)	C12—C11—Br1	118.3 (2)
N2—C2—H2	121 (2)	C11—C12—C17	118.4 (2)
N2—C3—N1	112.1 (2)	C11—C12—C13	123.5 (2)
N2—C3—H3	123.9	C17—C12—C13	118.1 (2)
N1—C3—H3	123.9	C14—C13—C12	121.0 (3)
C17—C4—C5	122.4 (2)	C14—C13—H13	119.5
C17—C4—N1	119.2 (2)	C12—C13—H13	119.5
C5—C4—N1	118.4 (2)	C13—C14—C15	120.8 (3)
C4—C5—C6	122.7 (2)	C13—C14—H14	119.6
C4—C5—C10	118.7 (2)	C15—C14—H14	119.6
C6—C5—C10	118.6 (2)	C16—C15—C14	120.5 (3)
C7—C6—C5	121.3 (3)	C16—C15—H15	119.7
C7—C6—H6	119.4	C14—C15—H15	119.7
C5—C6—H6	119.4	C15—C16—C17	120.9 (3)
C6—C7—C8	120.1 (3)	C15—C16—H16	119.6
C6—C7—H7	120.0	C17—C16—H16	119.6
C8—C7—H7	120.0	C4—C17—C16	122.3 (2)
C9—C8—C7	120.9 (3)	C4—C17—C12	119.1 (2)
C9—C8—H8	119.5	C16—C17—C12	118.6 (2)
C7—C8—H8	119.5	C3—N1—C1	105.9 (2)
C8—C9—C10	121.0 (3)	C3—N1—C4	128.0 (2)
C8—C9—H9	119.5	C1—N1—C4	126.1 (2)
C10—C9—H9	119.5	C3—N2—C2	105.1 (2)
C11—C10—C5	118.5 (2)		
			
N1—C1—C2—N2	0.4 (4)	C17—C12—C13—C14	1.2 (4)
C17—C4—C5—C6	−179.8 (2)	C12—C13—C14—C15	0.0 (4)
N1—C4—C5—C6	1.9 (4)	C13—C14—C15—C16	−1.0 (5)
C17—C4—C5—C10	1.1 (4)	C14—C15—C16—C17	0.8 (4)
N1—C4—C5—C10	−177.3 (2)	C5—C4—C17—C16	180.0 (2)
C4—C5—C6—C7	−179.8 (3)	N1—C4—C17—C16	−1.7 (4)
C10—C5—C6—C7	−0.6 (4)	C5—C4—C17—C12	−1.1 (4)
C5—C6—C7—C8	−1.1 (4)	N1—C4—C17—C12	177.2 (2)
C6—C7—C8—C9	1.9 (5)	C15—C16—C17—C4	179.4 (3)
C7—C8—C9—C10	−0.9 (4)	C15—C16—C17—C12	0.4 (4)
C4—C5—C10—C11	0.6 (4)	C11—C12—C17—C4	−0.5 (4)
C6—C5—C10—C11	−178.6 (2)	C13—C12—C17—C4	179.7 (2)
C4—C5—C10—C9	−179.3 (2)	C11—C12—C17—C16	178.5 (2)
C6—C5—C10—C9	1.5 (4)	C13—C12—C17—C16	−1.3 (4)
C8—C9—C10—C11	179.4 (3)	N2—C3—N1—C1	0.3 (3)
C8—C9—C10—C5	−0.8 (4)	N2—C3—N1—C4	−179.4 (2)
C5—C10—C11—C12	−2.2 (4)	C2—C1—N1—C3	−0.4 (3)
C9—C10—C11—C12	177.6 (3)	C2—C1—N1—C4	179.2 (3)
C5—C10—C11—Br1	175.01 (18)	C17—C4—N1—C3	95.3 (3)
C9—C10—C11—Br1	−5.1 (4)	C5—C4—N1—C3	−86.3 (3)
C10—C11—C12—C17	2.2 (4)	C17—C4—N1—C1	−84.3 (3)
Br1—C11—C12—C17	−175.05 (18)	C5—C4—N1—C1	94.1 (3)
C10—C11—C12—C13	−178.0 (2)	N1—C3—N2—C2	0.0 (3)
Br1—C11—C12—C13	4.8 (4)	C1—C2—N2—C3	−0.3 (4)
C11—C12—C13—C14	−178.7 (3)		

### Hydrogen-bond geometry (Å, º)

**Table:**

*D*—H···*A*	*D*—H	H···*A*	*D*···*A*	*D*—H···*A*
C3—H3···Br1^i^	0.95	2.86	3.687 (3)	147
C3—H3···N2^ii^	0.95	2.58	3.393 (4)	144

Symmetry codes: (i) −*x*+1, −*y*+2, −*z*+1; (ii) −*x*+1, −*y*+1, −*z*+2.
